# Risk of hyperprolactinemia and extrapyramidal symptoms in antipsychotic-treated schizophrenia: a retrospective single-center real-world study

**DOI:** 10.3389/fphar.2026.1884159

**Published:** 2026-09-07

**Authors:** Yong Shao, Zhe Lu, Yuyanan Zhang, Xueping Wang

**Affiliations:** 1 The Second Affiliated Hospital of Henan Medical University, Xinxiang, China; 2 Henan Collaborative Innovation Center of Prevention and Treatment of Mental Disorder, Xinxiang, China; 3 Brain Institute, Henan Academy of Innovations in Medical Science, Zhengzhou, China; 4 Peking University Institute of Mental Health, Peking University Sixth Hospital, Beijing, China; 5 NHC Key Laboratory of Mental Health (Peking University), Beijing, China; 6 National Clinical Research Center for Mental Disorders (Peking University Sixth Hospital), Beijing, China

**Keywords:** antipsychotics, EPS, hyperprolactinemia, real-world study, schizophrenia

## Abstract

Hyperprolactinemia (HPRL) is a common adverse reaction induced by antipsychotic therapy in patients with schizophrenia, which greatly undermines medication adherence and impairs physiological functions of multiple body systems. In this real-world study, we analyzed retrospective prolactin testing data to compare the incidence of HPRL between schizophrenia patients and patients with other psychiatric disorders, so as to identify clinical high-risk factors. Meanwhile, hormones including thyroid-stimulating hormone (TSH), free tetraiodothyronine (FT4), free triiodothyronine (FT3), tetraiodothyronine (T4), Triiodothyronine (T3), and estradiol that closely correlated with prolactin metabolism were also included in the analysis. The results indicated that female patients with schizophrenia had the highest risk of HPRL (19.31%, n = 2009), and women during the prime reproductive (25–40 years) showed the highest average prolactin concentration (1,389.22 ± 50.138 mIU/L, n = 966). Distinct antipsychotics usage rates were observed among different clinical subgroups. Risperidone, paliperidone and sulpiride showed an increasing trend with elevated PRL levels, whereas quetiapine and aripiprazole were more frequently prescribed in the low-risk HPRL group. Antipsychotics showed divergent dopamine D2/D3 and 5-HT2A receptor-binding affinities (Ki values), and we constructed a weighted multi-receptor affinity index that demonstrated a strong linear correlation with the PRL risk score (R^2^ = 0.878). Additionally, the average incidence of extrapyramidal symptoms (EPS) increased with rising PRL levels (*P* = 2.24E-10). Further analysis of EPS risk revealed distinct safety profiles across antipsychotics, with aripiprazole—characterized by low HPRL liability—demonstrating a higher EPS risk. The severe HPRL group presented a tendency of decreased thyroid function (TSH, *P* = 0.0027; FT4, *P* = 0.000031; FT3, *P* = 0.0076; T4, *P* = 0.0025) and a significant reduction in estradiol levels (*P* = 0.000049). Based on homogeneous clinical real-world data, this study comprehensively evaluated multiple antipsychotic medications simultaneously. It provides empirical clinical evidence for individualized medication strategies in patients with comorbid HPRL and EPS, and offers real-world evidence for the clinical early risk recognition, and long-term medication decision-making among patients with multiple adverse reactions concurrently.

## 1 Introduction

Prolactin is a key hormone that modulates human physiological functions (Bernard et al., 2015; Szukiewicz, 2024), and its abnormal elevation imposes well-documented short- and long-term detrimental effects (Bostwick et al., 2009; Milano et al., 2011). HPRL exerts pronounced adverse effects on sexual function, reproductive health, and skeletal system homeostasis (Solmi et al., 2023). Notably, HPRL is closely associated with infertility, affecting 15%–20% of women undergoing infertility evaluation and contributing to secondary amenorrhea (Haidenberg-David et al., 2024). HPRL impairs osteogenesis and reduces bone mineral density, thereby elevating the risk of skeletal fractures (di Filippo et al., 2020). Furthermore, long-term exposure to sustained HPRL increases the risk of breast cancer in women (Taipale et al., 2021).

HPRL is a highly prevalent adverse reaction to antipsychotic medications, with its incidence ranging from 30% to 80% among patients receiving antipsychotic treatment (Brandsma et al., 2021). Antipsychotics are the preferred treatment drugs for the symptomatic management of mental disorders, most notably schizophrenia. However, HPRL is strongly linked to poor medication adherence and disease recurrence (Milano et al., 2017). Given that chronic mental disorders such as schizophrenia typically require long-term maintenance pharmacotherapy, the adverse impacts of HPRL are likely to persist, resulting in prolonged harm to patients’ physical health. Thus, mitigating or preventing HPRL has become an essential consideration in clinical psychiatric pharmacotherapy. Current management strategies for antipsychotic-induced HPRL include dopamine agonists, selective estrogen receptor modulators, switching to lower-risk antipsychotics, and add-on therapy with third-generation antipsychotics (Montejo Á et al., 2017; Lu et al., 2022; Shim et al., 2007). Nevertheless, a recent study reported that switching from the original antipsychotic regimen to aripiprazole was associated with an increased risk of recurrence than other drugs (*P* < 0.001) in schizophrenia patients (Cai et al., 2023).

Extrapyramidal symptoms (EPS) constitute another common serious adverse effect of antipsychotic treatment. EPS constitute a severe factor compromising the adherence and safety of antipsychotic pharmacotherapy (Siafis et al., 2023; Krause et al., 2018). Epidemiological evidence indicates that within the first year of initiating treatment with atypical antipsychotics, 22.1% of patients develop new-onset EPS, with an overall EPS prevalence of up to 26.9% (Kadakia et al., 2022). Medical costs among patients with comorbid EPS are approximately twice as high as those without EPS. Although previous studies have demonstrated significant differences in EPS risk across antipsychotics, comparative data within homogeneous patient populations remain sparse, and conducting corresponding randomized controlled trials (RCTs) is associated with substantial costs. Furthermore, the multifaceted disruptive effects of antipsychotics may lead to additive adverse consequences. The overlapping comorbidity of common adverse drug reactions in daily clinical practice represents a critical challenge and bottleneck in the pharmacotherapy of schizophrenia. Real-world clinical studies employing large cohorts with refined subgroup analyses, while controlling for population homogeneity, offer a practical strategy for investigating actual drug effects. In recent years, real-world clinical research has emerged as a powerful tool with unique advantages (Kimura et al., 2024; Singh et al., 2018), especially in providing practical evidence for individualized clinical decision-making and improving the cost-effectiveness of research. In response to the aforementioned clinical challenges, the present study aimed to identify high-risk populations and investigate antipsychotic-related hyperprolactinemia through in-depth mining of clinical and laboratory data combined with medication trajectory analyses in specific patient cohorts. The effects of different second-generation antipsychotics on prolactin elevation were evaluated using real-world clinical data. Therefore, we identified high-risk subgroups for HPRL among patients with mental disorders, and analyzed the overall changes in EPS comorbidity as well as thyroid hormone and estrogen levels in patients with hyperprolactinemia. Specially, we examined medication prescribing patterns in patients with HPRL and EPS, and assessed the risk profiles of different antipsychotics.

## 2 Methods

### 2.1 Data sources and selection

The clinical test data were extracted from the Laboratory Information Management System (LIS) of the Second Affiliated Hospital of Henan Medical University. Prolactin test results of inpatients were retrieved from the database between January 2023 and December 2024. A retrospective analysis was performed on the medical records, including disease diagnosis, gender, and age of the included subjects.

In this study, subjects with any mental illness were classified as the mental illness patient group. Schizophrenia patients were included if they met the diagnostic criteria specified in the *ICD-10 Classification of Mental and Behavioral Disorders* (corresponding to DSM diagnostic criteria for schizophrenia). All clinical evaluations were performed by psychiatrists with at least 3 years of clinical experience. A total of 20,757 inpatients were enrolled, comprising 9,820 males and 10,991 females. Of these, 3,902 cases were diagnosed with schizophrenia, including 1,893 males and 2,009 females. All participants were independent individuals, and no duplicate cases were included in the study. We included patients with psychiatric diagnoses other than schizophrenia as a separate comparison group to investigate whether schizophrenia specific phenotypic characteristics exist. Post hoc power analysis was performed using G*Power to verify the robustness of the main findings, confirming that the sample size was sufficient to detect statistically significant effects.

### 2.2 Indicator testing

The Roche cobas e601 fully automated analyzer (manufacturer: Roche, Germany) was used in clinical testing. The reagents, calibrators, and quality control materials were Roche (manufacturer: Roche, Germany). The samples for hormone testing were collected at 6 a.m. Clinical test results of estradiol and thyroid-related hormones from the same batch of specific groups were included.

### 2.3 Data analysis

Based on the values of prolactin tests and referring to the expert consensus and the normal range set by laboratory itself, the normal reference range for prolactin is 86–324 mIU/L for men, and 102–496 mIU/L for women. The clinical practice guideline (Melmed et al., 2011) and multidisciplinary expert consensus defines the HPRL based on the prolactin concentration and the patient’s gender. The prolactin levels were divided into 5 groups: normal group (men ≤324 mIU/L, women ≤496 mIU/L), mild abnormality group (men 324–424 mIU/L, women 496–530 mIU/L), mild HPRL group (men 424 - 1060 mIU/L, women 530 - 1060 mIU/L), moderate HPRL group (1060 - 2120 mIU/L), and severe HPRL group (> 2120 mIU/L). The mild abnormality group was designated as a borderline category and excluded from the analysis of prolactin-related medication risk.

In the analysis of mild and severe HPRL groups, to clarify the effects of drug type and dosage on hyperprolactinemia, we reviewed medication records within a 21-day window (approximately 3 weeks) prior to the prolactin testing date. This timeframe was selected to capture steady-state drug exposure, as most antipsychotics reach steady-state plasma concentrations within 2–3 weeks of initiation or dose adjustment. Some patients underwent prolactin testing during the initial phase of hospitalization, which may have led to missing records of medication use. Only a small proportion of patients were diagnosed with first-episode schizophrenia and had not received prior pharmacotherapy. Pregnant and postpartum women were excluded from the analysis. Thyroid-related hormones and estradiol were measured in the same batch of samples as prolactin. The EPS phenotypes assessed included acute dystonia, akathisia, parkinsonism (drug induced parkinsonism), and tardive dyskinesia. In this study, we integrated case records and anticholinergic medication use for dual verification to ensure the reliability of phenotypic diagnoses.

In the calculation of risk values, *Difference* was defined as “*Difference* = drug usage rates of higher-risk group − lower-risk group”, while *Discrepancy* was defined as “*Discrepancy* = (higher-risk group − lower-risk group)/[(higher-risk group + lower-risk group)/2]”. Discrepancy can reflect the clinical utilization frequency of the corresponding antipsychotic. Risk assessment score was defined as “Risk assessment score = *Difference* × | *Discrepancy* |”. To enhance the reproducibility and generalizability of the prolactin risk scores derived from this study, we performed normalization on the raw calculated scores. The absolute value of the minimum score among the 10 antipsychotics was used as the reference unit (olanzapine, set to 1).

Ki values of antipsychotics were retrieved from the Psychoactive Drug Screening Program (PDSP) Ki Database (https://pdsp.unc.edu/databases/kidb.php) supported by the NIMH Psychoactive Drug Screening Program. Median Ki values from all human cloned receptor binding assays were calculated for each antipsychotic. Calculation methods and partial Ki data were retrieved from previous literature (Kaar et al., 2020). For drug risk internal validation, the dataset comprising 105 patients was derived from an independent case subset within the same batch, which had not been included in the initial risk discovery analysis.

### 2.4 Ethical consideration and privacy protection

This study is a retrospective observational study. The research protocol was reviewed by the Ethics Committee of the Second Affiliated Hospital of Henan Medical University. At the beginning of data extraction, the identifiable personal information of patients was anonymized. The research process adhered to the Helsinki Declaration and the ethical norms for human research in the country and region. All collected data were anonymized, and patient names, hospital numbers, and other direct identifiers were removed. Unique research codes were used instead. The database was encrypted and stored, and only authorized researchers had access.

## 3 Results

### 3.1 Risk of HPRL incidence in schizophrenia and other psychiatric disorders

Given the inherent significant sex-based differences in prolactin levels, all subjects were stratified by gender for subsequent statistical analysis. A comparison of prolactin levels between patients with schizophrenia and those with other mental disorders revealed a markedly higher proportion of severe HPRL in the schizophrenia group (Table 1). In both groups, females had a higher prevalence of severe and moderate HPRL than males; notably, the proportion of female schizophrenia patients (19.31%) with severe HPRL was approximately 5.38 times that of their male counterparts.

**TABLE 1:** Incidence differences of hyperprolactinemia in schizophrenia vs. other psychiatric disorders.

Group	SCZ		Other	
	Men (n = 1893)	Women (n = 2009)	Men (n = 7927)	Women (n = 8982)
Severe HPRL	**3.59%**	**19.31%**	**1.55%**	**10.33%**
Moderate HPRL	17.49%	20.06%	9.16%	15.98%
Mild HPRL	40.25%	23.20%	39.57%	28.68%
Mild abnormality	9.72%	2.09%	15.34%	3.19%
Normal	28.95%	35.34%	34.38%	41.82%

Bold values indicate the subgroup with the largest gender difference ratios.

HPRL exerts a considerable impact on individuals of reproductive age. Given the elevated risk of HPRL in patients with schizophrenia, we analyzed prolactin levels across distinct age groups. Among all participants, prolactin levels peaked during the prime reproductive period (25–40 years) in female schizophrenia, presenting the maximum mean prolactin concentration of 1,389.22 ± 50.138 mIU/L (n = 966, Table 2). Notably, female patients with schizophrenia had higher mean prolactin levels than those with other mental disorders across all age groups, reflecting their elevated susceptibility to HPRL and a greater risk of associated adverse effects.

**TABLE 2:** Age-stratified prolactin levels in schizophrenia and other psychiatric disorders.

Group	SCZ woman			SCZ man			Other woman			Other man			*P*-value SCZ vs. other	
Age (years)	Mean	SE	n	Mean	SE	n	Mean	SE	n	Mean	SE	n	Woman	man
≤18	1217.56	119.714	104	844.62	62.177	113	860.72	16.916	2372	625.74	12.483	1571	0.049	5.6E-05
18-25	1287.36	70.418	331	768.96	27.242	481	1174.00	34.174	1297	636.04	13.086	1509	0.13	4.9E-05
25-40	1389.22	50.138	966	710.38	23.546	941	1138.63	28.980	2131	531.07	9.684	2429	0.00022	1.7E-14
40-60	1235.69	62.009	551	609.59	33.054	336	895.58	24.546	2221	459.06	8.536	1860	5.3E-07	0.0001
>60	926.85	137.322	57	557.15	72.185	22	766.56	36.039	961	493.45	17.022	558	0.13	0.19

### 3.2 Drug usage rate differences between HPRL subgroups in female schizophrenia patients

To investigate the impact of antipsychotic use on prolactin levels, we analyzed medication-related differences between patients with mild and severe HPRL. Antipsychotic types and dosages were extracted from drug prescriptions in electronic medical records. Due to the limited sample size of male cases, available clinical data was too little for intergroup comparisons.

**FIGURE 1:**
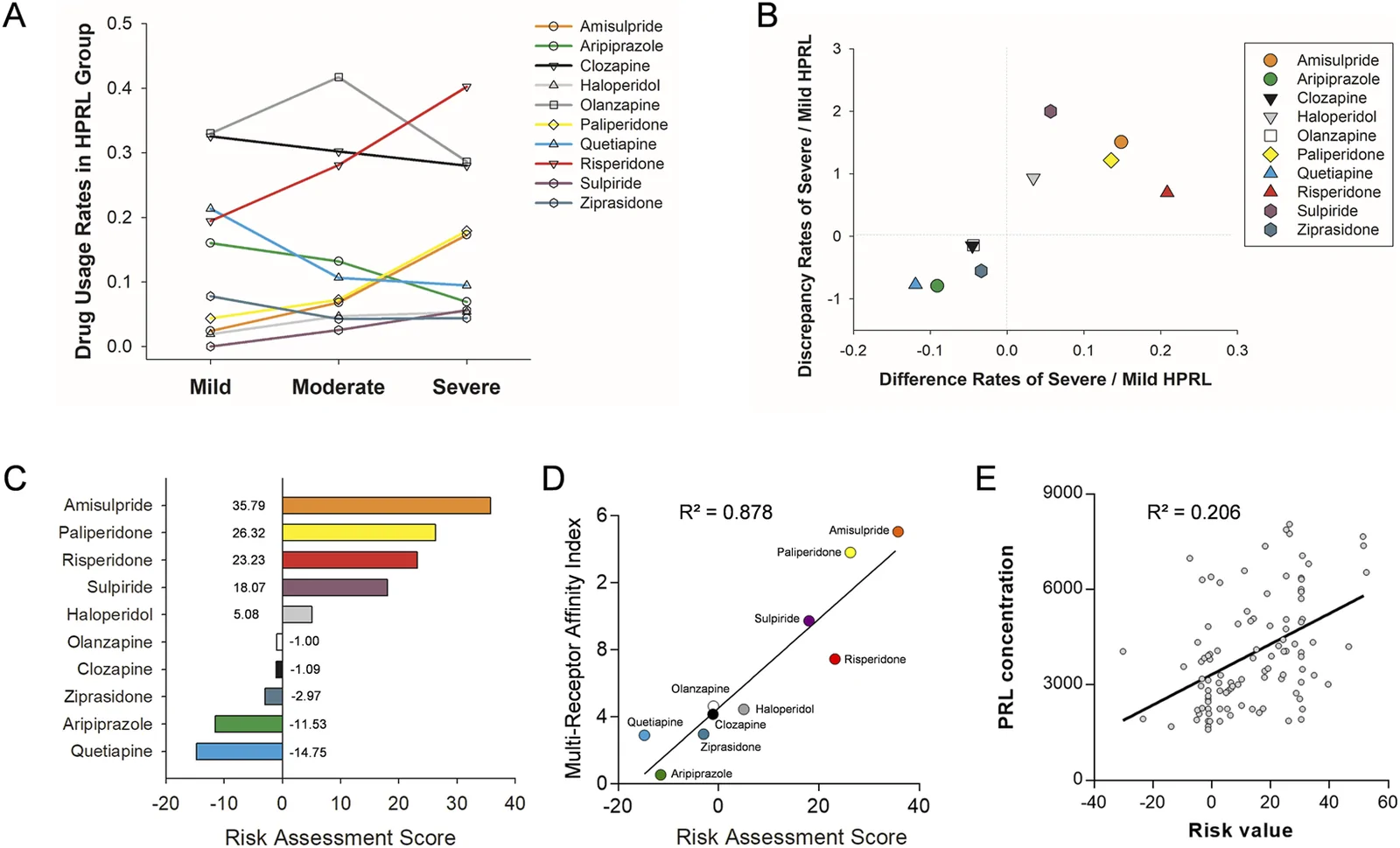
Relationship between drug usage and prolactin levels in female schizophrenia patients stratified by HPRL severity. **(A)** Frequency of antipsychotic use in Mild (n = 206), Moderate (n = 235), and Severe (n = 318) HPRL patients. **(B)** Risk effect scatter plot of 10 antipsychotics associated with HPRL severity, comparing Mild HPRL and Severe HPRL. “*Difference* = drug usage rates of higher-risk group − lower-risk group”, and “*Discrepancy* = (higher-risk group − lower-risk group)/[(higher-risk group + lower-risk group)/2]”. **(C)** The risk assessment scores for 10 antipsychotic drugs. Raw prolactin risk scores were normalized,and the minimum absolute value of olanzapine among 10 antipsychotics was set as reference (value = 1), and the value is marked beside the column. **(D)** Linear correlation analysis between the multi-receptor affinity index (calculated comprehensively based on Ki values of D2, D3 and 5-HT2A receptors) and drug prolactin risk assessment score. R^2^ = 0.878, slope significance: *P* < 0.0001. **(E)** Predictive effects of 10 antipsychotics in the validation dataset (n = 105). R^2^ = 0.205, slope significance: *P* < 0.0001.

As shown in Figure 1A, drug usage rates were compared across three groups with different PRL levels. With the exception of olanzapine, which was the most commonly prescribed antipsychotic on average, the usage rates of the remaining drugs exhibited consistent trends across the three groups. A pronounced increasing trend in usage frequency was observed for risperidone, amisulpride, paliperidone, and sulpiride with rising prolactin levels. By contrast, quetiapine, aripiprazole, clozapine, and ziprasidone showed lower prescription rates. Furthermore, we compared drug usage difference and discrepancy between the mild and severe HPRL groups to estimate the risk of different antipsychotics, as illustrated in Figure 1B. The severe HPRL group received a slightly higher dosage of risperidone but lower dosages of quetiapine and aripiprazole (Supplementary Table S1). We also performed statistical analysis of PRL levels in patients receiving monotherapy (Supplementary Figure S1), to further clarify the independent pharmacological effect of antipsychotics on prolactin secretion.

Subsequently, the HPRL risk assessment scores were analyzed (Figure 1C) and further validated using drug receptor affinity parameters. Antipsychotics displayed divergent receptor-binding affinity, as reflected by Ki values (lower Ki represents stronger receptor binding ability, Supplementary Table S2). We thereby constructed a weighted multi-receptor affinity index covering three core pharmacological targets, including dopamine D2, D3 and 5-HT2A receptors. Relevant formula was presented as: Multi-Receptor Affinity Index = (Ki_D3_/Ki_D2_)^2^ + Ki_5-HT2A_/600. Linear regression analysis confirmed a favorable linear correlation between this receptor affinity index and clinically derived PRL risk assessment score (R^2^ = 0.878, Figure 1D). For validation, risk assessment scores were applied, and antipsychotic medication use and corresponding dosages among female patients with schizophrenia (n = 105) were incorporated as weighting coefficients in the calculation of risk values. Results are presented in Figure 1E, with an R^2^ of 0.20 and *P* < 0.0001.

### 3.3 Synergistic increase in EPS risk in patients with hyperprolactinemia

**FIGURE 2:**
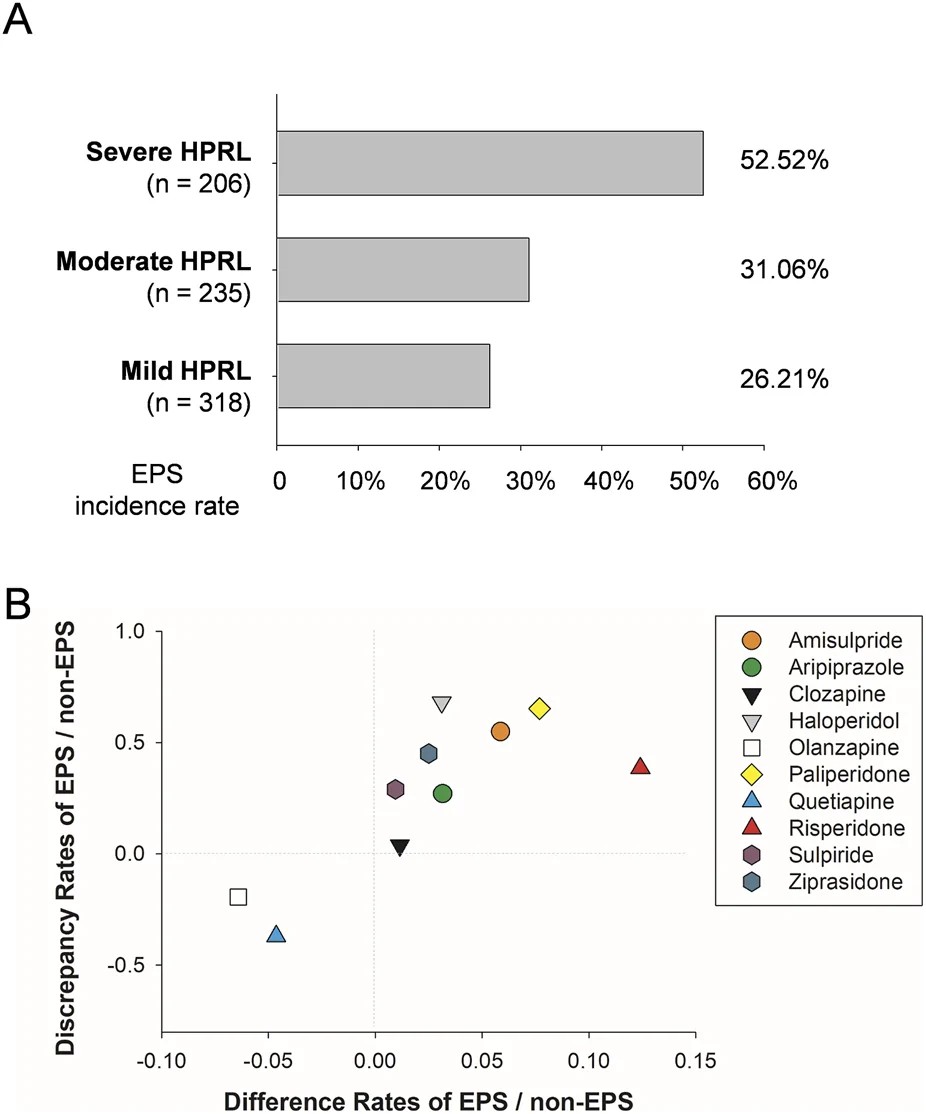
Risk of extrapyramidal symptoms according to hyperprolactinemia level and antipsychotic medication. **(A)** Comparisons of the incidence of EPS among different HPRL groups. Cochran-Armitage trend test, Z = 6.34, *P* = 2.24E-10. **(B)** Risk effect scatter plot of 10 antipsychotics for the occurrence of EPS. Across all hyperprolactinemia groups, 294 participants developed EPS and 465 did not.

Prolactin elevation is closely associated with the blockade of central hypothalamic dopamine D2 receptors by antipsychotic medications. With increasing severity of hyperprolactinemia, the incidence of extrapyramidal symptoms (EPS) showed a gradual and stepwise rise across different HPRL subgroups at the overall population level (Cochran-Armitage trend test, Z = 6.34, *P* = 2.24E-10). As shown in Figure 2A, the incidence of EPS in the severe HPRL group was approximately twice that in the mild HPRL group.

We then analyzed the usage frequency of individual antipsychotics in female HPRL patients stratified by whether EPS occurs or not, and visualized the differences in Figure 2B. Among all medications, haloperidol, amisulpride, paliperidone, and risperidone were associated with a higher risk of EPS compared with other drugs, whereas quetiapine exhibited the largest negative discrepancy value (Supplementary Table S3). Notably, although aripiprazole showed a prolactin-lowering effect in previous analyses, it was associated with an increased risk of EPS in the present study. Further separate analysis of aripiprazole group (n = 54) revealed no significant differences in aripiprazole dosage or PRL levels between the EPS and non-EPS subgroups (Supplementary Table S4). However, subgroup analysis of aripiprazole dosage (Supplementary Table S5) showed that patients receiving high-dose aripiprazole (15–30 mg daily) exhibited significantly (*P* = 0.0118) lower PRL concentrations compared with those administered low-dose aripiprazole (<15 mg daily). Proportions of patients with concomitant use of anticholinergics, mood stabilizers, and benzodiazepines are provided in the Supplementary Table S6. Given the risk of excessively small group sizes with further stratification, no additional multiple subgroup analyses were performed in this study.

### 3.4 Prolactin, thyroid and estradiol relationships in female schizophrenia patients with HPRL subgroups

**FIGURE 3:**
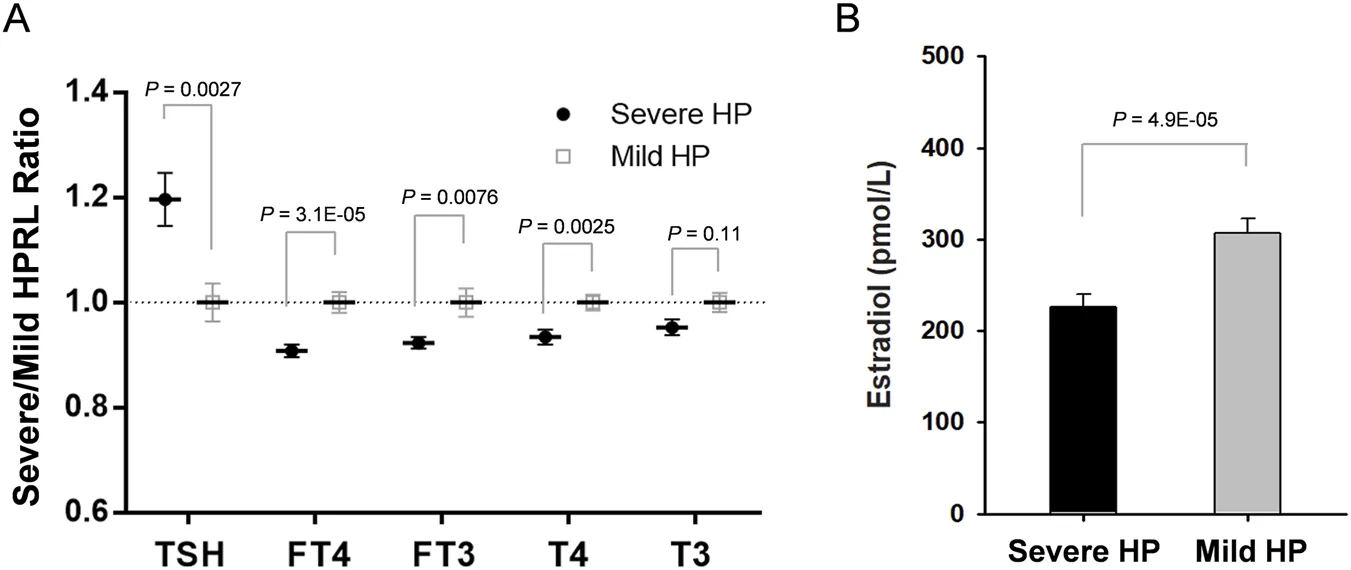
The prolactin, thyroid and estradiol relationships in female schizophrenia patients with HPRL. **(A)** Comparisons of TSH, FT4, FT3, T4, and T3 levels in Mild HPRL (n = 311) vs. Severe HPRL (n = 245) patients. Ratio of severe HPRL to mild HPRL; mean value of mild HPRL group set as reference = 1. Statistical significance between groups is marked on the figure; **(B)** Estradiol levels in Mild HPRL (n = 400) and Severe HPRL (n = 371) patients. One-way ANCOVA adjusting for age showed a statistically significant difference in estradiol levels between the two groups (*F* = 16.90, *P* = 0.000049).

Prolactin and thyroid hormone secretion are both regulated by the hypothalamic-pituitary axis, and thyrotropin-releasing hormone (TRH) can promote prolactin secretion. To clarify the associations among these hormones in patients with HPRL, intergroup statistical analyses were performed for thyroid-related hormones and estradiol. Female patients with mild HPRL and severe HPRL were enrolled for comparative analysis. For ease of comparing thyroid-related hormone levels, data from the Mild HPRL group were normalized. The results demonstrated that, compared with the Mild HPRL group, the Severe HPRL group had a significant increase in thyroid-stimulating hormone (TSH) levels (Figure 3A), accompanied by significant decreases in free tetraiodothyronine (FT4), free triiodothyronine (FT3), and tetraiodothyronine (T4). In the severe HPRL group, there was an overall trend of decreased thyroid function.

Estrogen and prolactin exhibit a close and intricate physiological connection in the human body. Estradiol concentrations were significantly lower in the Severe HPRL group (Figure 3B). Age was included as a covariate in the between-group comparison, and the difference remained statistically significant (*P* = 0.000049). Notably, no significant age difference was observed between the two groups (Supplementary Table S7).

## 4 Discussion

This study identified high-risk subgroups and age-related characteristics of HPRL in patients with schizophrenia, elucidated the association of antipsychotics with HPRL induction or inhibition, characterized the risk profiles of different antipsychotic medications with regard to the occurrence of HPRL and EPS, and further delineated thyroid hormone and estradiol profiles in female patients with HPRL.

The degree of prolactin elevation varied substantially by age and gender. Notably, the age group with the highest prolactin levels overlaps with the primary reproductive period (Table 2), emphasizing HPRL’s potential impact on female reproductive health. In males, HPRL is a key contributor to hypogonadism (Haidenberg-David et al., 2024), which also impairs male reproductive health. Prolactin directly modulates bone metabolism, and HPRL impairs osteogenesis and reduces bone mineral density, thereby increasing fracture risk (di Filippo et al., 2020). In the present study, severe HPRL patients exhibited significantly lower estradiol levels (Figure 3B). Estrogen maintains bone remodeling balance by regulating osteoblast and osteoclast activity, with a pronounced effect on osteoporosis in the aging population (González-Rodríguez et al., 2020). Age-related estrogen decline plus antipsychotic-induced HPRL increases skeletal adverse event risks (e.g., fractures), which can be life-threatening in the elderly. Moreover, accumulating evidence suggests potential oncogenic risks: Johnston et al. demonstrated that risperidone can activate signal transducer and activator of transcription 5 (STAT5), inhibit cell apoptosis, and promote the progression of precancerous lesions to malignant tumors (Johnston et al., 2018), whereas aripiprazole does not exert such effects. This is particularly critical for mental disorder patients with pre-existing precancerous lesions, and implies that HPRL may harbor uncharacterized long-term and potential hazards that warrant further investigation. Routine prolactin testing is recommended in patients on high-risk PRL-elevating medications. While macroprolactin may interfere with some prolactin test results (Brandsma et al., 2021), it barely affects the classification of HPRL severity (Ruljancic et al., 2021), indicating the reliability of prolactin testing for clinical HPRL evaluation.

Intergroup difference analyses convincedly indicated that risperidone, amisulpride, paliperidone, and sulpiride carry a higher risk of inducing prolactin elevation, while quetiapine and aripiprazole are associated with a lower risk. Antipsychotics with dopamine D2 receptor antagonistic activity disrupt the inhibitory regulation of prolactin secretion by blocking dopamine receptors, thereby triggering HPRL. Aripiprazole, by virtue of its partial agonist activity at D2 receptors, exerts a prolactin-lowering effect. Previous meta-analysis had shown that high-dose aripiprazole can significantly reduce prolactin levels compared with low-dose (Lin et al., 2025). Results from a randomized controlled trial on antipsychotic monotherapy revealed that aripiprazole and quetiapine are associated with a lower risk of HPRL, while risperidone and amisulpride carry a higher risk (Zhao et al., 2026). Our real-world clinical data findings were consistent with these reports. Previous studies have shown that EPS is associated with dopamine D_2_ receptor occupancy, with substantial variability in risk magnitude across different antipsychotics (Siafis et al., 2023). Meta-analyses have indicated that nearly all antipsychotics may induce dose-dependent EPS, whereas quetiapine showed no association with EPS even at high doses (Siafis et al., 2023). In another study focusing on EPS-vulnerable patients, clozapine and quetiapine were recommended as more favorable antipsychotics (Friedman, 2003). A more recent analysis reported that haloperidol, risperidone, and quetiapine carried a higher risk of EPS compared with placebo (Krause et al., 2018), and aripiprazole was associated with a higher EPS risk than quetiapine. Prior pharmacogenetic studies have also indicated the pivotal role of candidate genes in aripiprazole related movement adverse reactions (Wang et al., 2023). Therefore, we cannot rule out that genetic factors may mediate the association between aripiprazole and EPS susceptibility.

Blockade of D_2_ receptors in the nigrostriatal dopaminergic pathway impairs movement regulation and causes EPS, whereas inhibition in the tuberoinfundibular pathway disrupts hypothalamic-pituitary endocrine homeostasis, leading to HPRL (Siafis et al., 2023; Kaar et al., 2020; Yoshida and Takeuchi, 2021; Haddad and Sharma, 2007). This region-dependent mechanism underlies the distinct adverse effect profiles of antipsychotics. Inter-drug and inter-individual differences in adverse reaction risk cannot be attributed to D_2_ blockade alone. Using composite proportional and weighted calculations, we established the quantitative Ki relationship of dopamine D2, D3 and 5-HT_2_A receptors across the 10 included antipsychotics, which exhibited a strong linear correlation with our PRL risk score (R^2^ = 0.878). Nevertheless, refined analyses focusing on the intrinsic efficacy of individual antipsychotics are still warranted. Given that the effects of other antipsychotic medications (e.g., risperidone and aripiprazole) on prolactin have been relatively well characterized (Kearns et al., 2000; Preda and Shapiro, 2020), here we focus on exploring the potential mechanism underlying quetiapine’s low HPRL risk. Meta-analysis found the risk of hormone elevation induced by quetiapine is negligible (Lin et al., 2025). Quetiapine exhibits a short duration of D2 receptor occupancy, leading to transient and mild interference with dopamine signaling (Mushtaq et al., 2012; Atmaca et al., 2002; Keller and Mongini, 2002). Furthermore, it rapidly crosses the blood-brain barrier, minimizing prolonged D2 receptor blockade in the pituitary gland and consequently reducing the propensity to disrupt prolactin homeostasis. Beyond its D2 receptor-related properties, quetiapine potently blocks 5-hydroxytryptamine (5-HT2A) receptors (Jones et al., 2001). Since 5-hydroxytryptamine promotes prolactin release, the blockade of 5-HT2A receptors by quetiapine may contribute to a reduction in prolactin levels to a certain extent.

In a small sample of female patients with depression who developed severe HPRL under concomitant risperidone administration, we compared their PRL levels with those in the severe HPRL subgroup of patients with schizophrenia. No significant between-group difference was observed (*P* = 0.74; depression: mean ± SE = 3,970.32 ± 239.04, n = 44; schizophrenia: mean ± SE = 3,881.96 ± 119.16, n = 163). Thus, our findings on antipsychotic medications also offer extrapolatable reference value for patients with other disorders receiving concomitant antipsychotic therapy. The effects of antipsychotic medications across different psychiatric disorders warrant further in-depth and specific investigation.

A delicate balance exists among prolactin, estradiol, and thyroid-related hormones in the endocrine system, with mutual regulatory effects between these factors. HPRL can lead to estrogen deficiency, a phenomenon speculated to be attributed to antipsychotic exposure or stress responses (Brand et al., 2021). Elevated prolactin levels inhibit the secretion of hypothalamic gonadotropin-releasing hormone secretion, result in estrogen reduction. Notably, estrogen deficiency is also regarded as a contributor to diminished antipsychotic efficacy, which in turn exacerbates psychiatric symptoms (González-Rodríguez et al., 2022). Studies have indicated that antipsychotic treatment can alter patients’ thyroid hormone levels. In schizophrenia patients after initiation of antipsychotic therapy, the incidence of hypothyroidism was significantly higher than that in the control group, and this intergroup difference was absent prior to treatment initiation (Melamed et al., 2020). A case report demonstrated that one patient exhibited a significant elevation in thyroid-stimulating hormone (TSH) levels and a mild reduction in free thyroxine (FT4) levels following paliperidone administration (Mohammed et al., 2025). This is consistent with our observations in the severe HPRL group, that paliperidone was more frequently used and a corresponding trend in thyroid-related hormone levels was identified. Previous study also revealed that patients receiving antipsychotic treatment have a higher prevalence of sexual dysfunction, which is associated with elevated prolactin levels and subclinical hypothyroidism (Zhang et al., 2018). Consistent with these findings, female schizophrenia patients in the severe HPRL subgroup in our study exhibited decreased average estrogen levels, accompanied by alterations in thyroid hormone profiles.

Limitations of the present study are as follows. Firstly, as an observational study based on real-world data, this study cannot draw causal inferences. Secondly, this study only includes the examinations and medical records of inpatients at a single center, which may not reflect the conditions of outpatients with milder symptoms. Lastly, the model achieved an R^2^ of 0.20 in the validation dataset, showing limited predictive performance. This may be confounded by baseline medication regimens, treatment duration, and concomitant medications, restricting the generalizability of the results. Further external validation is warranted.

To summarize, this real-world study provides valuable references for guiding clinical intervention strategies. By integrating patients’ actual medications, we identified disparities in the utilization frequency of various antipsychotics across different subgroups. This study demonstrates that real-world research can complement traditional RCTs, which are costly, use strict inclusion criteria, and often exclude severely ill patients for feasibility and ethical reasons. Therefore, integrating antipsychotics information to select optimal pharmacotherapeutic strategy at the initiation of treatment is crucial for managing adverse reactions in schizophrenia patients at high risk of HPRL.

## Data Availability

The data analyzed in this study is subject to the following licenses/restrictions: The original clinical data cannot be shared, but one can apply for the re-analysis of the classified statistical data. Requests to access these datasets should be directed to XW, xuepingwang@bjmu.edu.cn.
