# Supplementary material for: Risk of hyperprolactinemia and extrapyramidal symptoms in antipsychotic-treated schizophrenia: a retrospective single-center real-world study
Source: Front Pharmacol. 2026 Sep 7;17:1884159. doi: 10.3389/fphar.2026.1884159 (PMC13590559; doi:10.3389/fphar.2026.1884159)
Supplement: Supplementary file 1 [file Supplementaryfile1.docx]

**SUPPLEMENTARY TABLES**

**Table S1. Analysis of dosages of major drugs with differential risks in HPRL across different groups.**

| Group | Drug |  | PRL concentration (mIU/L) | | |  | Drug dose (mg) dose | | |
| --- | --- | --- | --- | --- | --- | --- | --- | --- | --- |
|  |  |  | mean | SD | n |  | mean | SD | *P* |
| Severe HP | Risperidone |  | 3617.86 | 1529.27 | 149 |  | 4.21 | 1.59 | *P* = 0.12 |
| Mild HP | Risperidone |  | 769.27 | 153.59 | 41 |  | 3.74 | 1.66 |  |
|  |  |  |  |  |  |  |  |  |  |
| Severe HP | Quetiapine |  | 3732.22 | 1395.34 | 27 |  | 361.11 | 253.09 | *P* = 0.50 |
| Mild HP | Quetiapine |  | 699.62 | 114.96 | 44 |  | 397.73 | 244.23 |  |
|  |  |  |  |  |  |  |  |  |  |
| Severe HP | Aripiprazole |  | 3616.00 | 1561.27 | 24 |  | 10.88 | 6.30 | *P* = 0.021 |
| Mild HP | Aripiprazole |  | 731.31 | 145.81 | 30 |  | 15.67 | 7.85 |  |
|  |  |  |  |  |  |  |  |  |  |
| Severe HP | Paliperidone* |  | 4827.36 | 2087.81 | 50 |  | 5.58 | 2.35 | NA |
|  |  |  |  |  |  |  |  |  |  |
| Severe HP | Amisulpride* |  | 4472.20 | 1876.31 | 56 |  | 484.82 | 233.92 | NA |

* Drug subgroups with small sample sizes were excluded from statistical analyses.

**Table S2. Median Ki values of different antipsychotics.**

| Drug | **D2** | **D3** | **5-HT2A** |
| --- | --- | --- | --- |
| Haloperidol | 1.4 | 2.9 | 84.7 |
| Clozapine | 119 | 242 | 6.5 |
| Olanzapine | 20.4 | 43.8 | 3.2 |
| Quetiapine | 212.5 | 340 | 200 |
| Risperidone | 2.2 | 6 | 0.4 |
| Amisulpride | 2.2 | 2.4 | 8304 |
| Aripiprazole | 1.4 | 1 | 8.7 |
| Ziprasidone | 5 | 8.6 | 0.7 |
| Paliperidone | 0.7 | 2.6 | 1.11 |
| Sulpiride | 7.9 | 10.4 | 4786 |

**Table S3.** **Antipsychotic use frequency in female schizophrenia patients with EPS vs. non-EPS.**

| Drug* | EPS (n = 294) |  | non-EPS (n = 465) |  | Difference | Discrepancy |
| --- | --- | --- | --- | --- | --- | --- |
| Aripiprazole | 13.27% |  | 10.11% |  | 3.16% | 27.02% |
| Clozapine | 30.61% |  | 29.46% |  | 1.15% | 3.83% |
| Haloperidol | 6.12% |  | 3.01% |  | 3.11% | 68.14% |
| Amisulpride | 13.61% |  | 7.74% |  | 5.86% | 54.93% |
| Olanzapine | 29.93% |  | 36.34% |  | -6.41% | -19.35% |
| Paliperidone | 15.65% |  | 7.96% |  | 7.69% | 65.15% |
| Quetiapine | 10.20% |  | 14.84% |  | -4.63% | -37.01% |
| Risperidone | 38.44% |  | 26.02% |  | 12.41% | 38.52% |
| Ziprasidone | 6.80% |  | 4.30% |  | 2.50% | 45.06% |
| Sulpiride | 3.74% |  | 2.80% |  | 0.95% | 28.94% |

*Combination therapy with two or more antipsychotic agents is common in inpatient psychiatric treatment. Patients receiving antipsychotic monotherapy were also analyzed in Supplementary Figure S1

**Table S****4** Quantitative analysis of EPS in the aripiprazole-treated group.

| Item | Group | n | Mean | Std.Dev | Std.Err | *P*-value |
| --- | --- | --- | --- | --- | --- | --- |
| Aripiprazole dose | EPS | 23 | 13.48 | 7.60 | 1.59 | 0.840 |
|  | non-EPS | 31 | 13.90 | 7.66 | 1.38 |  |
|  |  |  |  |  |  |  |
| PRL concentration | EPS | 23 | 2066.22 | 1593.24 | 332.21 | 0.853 |
|  | non-EPS | 31 | 1974.20 | 1929.39 | 346.53 |  |

**Table S5** Quantitative analysis of PRL levels in the aripiprazole-treated group.

| Item | Dose grouping * | n | Mean | Std.Dev | Std.Err | *P*-value |
| --- | --- | --- | --- | --- | --- | --- |
| Aripiprazole dose value | Low | 32 | 8.31 | 2.63 | 0.47 | NA |
|  | High | 22 | 21.59 | 4.97 | 1.06 |  |
|  |  |  |  |  |  |  |
| PRL concentration | Low | 32 | 2510.38 | 1983.27 | 350.60 | **0.0118** |
|  | High | 22 | 1290.50 | 1119.06 | 238.58 |  |

*Patients receiving aripiprazole at a daily dose < 15 mg were categorized into the low-dose group, and those with a daily dose of 15–30 mg were classified into the high-dose group. No patients received a daily aripiprazole dose exceeding 30 mg.

**Table S6** Proportion of Patients with Concomitant Use of Anticholinergics, Mood Stabilizers, and Benzodiazepines

| Drug category | Number of drug users | Total sample size | Proportion |
| --- | --- | --- | --- |
| Anticholinergics | 249 | 759 | 32.81% |
| Mood Stabilizers | 143 | 759 | 18.84% |
| Benzodiazepines | 220 | 759 | 28.99% |

**Table S7. Mean age statistics of female HPRL patients with estradiol data in two groups.**

| Group | Mean | Std.Err | n | *P** |
| --- | --- | --- | --- | --- |
| Mild HPRL | 34.69 | 0.60 | 400 | 0.285 |
| Severe HPRL | 33.78 | 0.60 | 371 |  |

* Student’s T-test

**SUPPLEMENTARY FIGURES**

**Figure S1** PRL concentrations in female patients with schizophrenia receiving single antipsychotic monotherapy.
